# Carving a niche for immunotherapy in ovarian cancer

**DOI:** 10.18632/oncotarget.27864

**Published:** 2021-01-05

**Authors:** Nitasha Gupta, Erika Lampert, Jung-Min Lee

**Keywords:** immunotherapy, PARP inhibitor, ovarian cancer, combination therapy

Ovarian cancer is one of the most aggressive women’s cancers and is difficult to treat in its advanced stages, where tumor recurrence and chemotherapy resistance are commonplace. As such, clinical trials with novel drug combination strategies including PARP inhibitors and immune checkpoint blockade (ICB) have proliferated to address this unmet clinical need. A recent study published in *Clinical Cancer Research* by Lampert *et al*. describes the efficacy, tolerability, and biomarker evaluation from a proof-of-concept phase II trial of the anti–PD-L1 antibody durvalumab and a PARP inhibitor olaparib in 35 patients with recurrent ovarian cancer based on positive safety data and preliminary antitumor activity from a phase I trial [1, 2]. Such drug combinations have been hypothesized to leverage synthetic lethality as well as the tumor milieu to provide greater antitumor activity than monotherapy alone. More specifically, PARP inhibitors kill tumor cells by blocking base excision repair and trapping PARP enzymes at damaged DNA, resulting in DNA breaks and cytotoxicity, particularly in cells with underlying homologous recombination deficiency (e.g., *BRCA1* and *BRCA2* mutations) [3]. Also, PARP inhibition has been shown preclinically to enhance PD-L1 expression and induce STING pathway activation, in addition to stimulating IFNγ and other inflammatory cytokines, promoting a net immunostimulatory environment [4, 5]. Thus, it was hypothesized that PARP inhibition would enhance the clinical response to ICB by producing a more immunogenic tumor microenvironment (TME).

Lampert *et al*. tested this hypothesis in ovarian cancer patients by evaluating clinical activity of combination durvalumab and olaparib, as well as investigating comprehensive correlative endpoints using tissues and blood samples. Moreover, it was the first ICB trial in ovarian cancer to collect fresh pre- and on-treatment tumor core biopsies, enabling a more accurate representation of the dynamic immune TME than archival tumor samples from primary debulking surgery or biopsies at first recurrence. In this study, neither baseline nor changes in PD-L1 expression were associated with clinical response. This result is somewhat at odds with preliminary findings in less heavily pretreated populations, requiring further prospective validation. For instance, post-hoc subgroup analysis from JAVELIN-200 (48% with 1 prior line of therapy, 52% with 2–3 prior lines of therapy), a randomized phase III trial looking at the anti–PD-L1 antibody avelumab in combination with pegylated liposomal doxorubicin (PLD), examined 442 patients with evaluable PD-L1 expression (78% of the total study population). Patients with PD-L1–positive tumors in the combination arm had a longer progression-free survival (PFS) than those in the PLD arm (3.7 [95% CI 2.2–5.6] vs. 1.9 months [95% CI 1.9–3.6]; *p* = 0.0048), while patients in the PD-L1–positive subgroup of the avelumab arm did not demonstrate longer PFS relative to those within the PLD arm [6]. Similarly, KEYNOTE-100 (76% ≤ 2 prior therapies, 24% 3–5 prior therapies), a single-arm phase II trial evaluating pembrolizumab monotherapy, reported a trend toward increased objective response rate (ORR) with a higher combined positive score (CPS) of tumor and immune PD-L1 staining (13.8% [95% CI 6.5–24.7] in CPS ≥ 10 vs. 7.3% [95% CI 3.4–13.3] in CPS < 1; *p* = ns), although overall ORR in all-comers was still modest at 8.5% (95% CI 5.9–11.8) [7, 8].

Though preclinical studies suggest that PARP inhibition induces STING pathway activation and increases tumor mutational burden (TMB), Lampert *et al*. reported no significant changes in TMB or STING expression with treatment, suggesting that both mechanisms are unlikely drivers of clinical response to ICB, consistent with findings in other cancers as well [4, 9, 10]. Optimal measurement of STING pathway activation is challenging, and the clinical value of its measurement is to be determined. Instead, Lampert *et al*. found that treatment induced several immunostimulatory changes, including increased mRNA expression of *IFNγ* and IFNγ-induced chemokines *CXCL9* and *CXCL10*; systemic production of IFNγ and TNFα; and tumoral infiltration by lymphocytes. Enhanced plasma IFNγ protein levels were associated with response and improved PFS. Baseline expression of an IFNγ-related gene signature by RNA-seq analysis was also associated with clinical benefit. Similar findings were observed in TOPACIO, in which mutational signature 3 and IFN-signaling in the CD8+ T-cell compartment of the TME were identified as possible biomarkers of clinical response to niraparib plus pembrolizumab [11, 12].

Notably, Lampert *et al*. found an increase in angiogenic factors (e.g., VEGFR3) on-treatment, which may have contributed to the poor ORR through immunosuppression by activation of myeloid-derived suppressor cells and regulatory T cells, while reducing the activity of antigen-presenting cells and effector T cells [13]. This finding is supportive of triplet approaches including PARP inhibitors, ICB, and VEGF/VEGFR inhibitors. A phase I/II study combining olaparib, durvalumab, and the VEGFR1–3 inhibitor cediranib in recurrent ovarian cancer is ongoing (NCT02484404), and a multicenter randomized phase II trial comparing triplet therapy with standard-of-care chemotherapy and other targeted drugs in platinum-resistant ovarian cancer is opening in 2021 (NRG GY-023).

The study by Lampert *et al*. adds to a growing list of ICB plus PARP inhibitor or chemotherapy clinical trials in ovarian cancer in which ORR (complete response [CR] + partial response [PR]) is more modest than expected if the drugs were working synergistically (Table 1). That said, ORR per Response Evaluation Criteria in Solid Tumors (RECIST) may not always be the optimal indicator of clinical benefit depending on the underlying cancer and therapeutic agents used. For instance, ICB may display atypical response patterns, such as delayed tumor size reduction, mixed response, or an initial tumor burden increase due to pseudoprogression [14]. MEDIOLA is the exception with an ORR of 72%, likely due to its platinum-sensitive, PARP inhibitor–naïve, *BRCA*-mutant population who is intrinsically more responsive to PARP inhibition alone [15]. The 71% disease control rate (DCR; CR + PR + stable disease [SD]) and 34% clinical benefit rate (PR + SD ≥ 6 months) Lampert *et al*. reported is therefore notable, especially given the nature of this difficult-to-treat population (86% platinum-resistant, 52% with ≥ 4 prior therapies, 77% *BRCA* wild type).

**Table 1:** Recently published or reported studies evaluating the combination of ICB with other agents in ovarian cancer

Study	Disease	Immunotherapy	Combined with	ORR/DCR, % (95% CI)	PFS/OS, mos (95% CI)
TOPACIO NCT02657889 Single arm Phase I/II Konstantinopoulos *et al*. 2019 [11]	60 evaluable patients Recurrent setting 30 platinum-resistant 17 platinum-refractory 15 platinum-toxicity/allergy 49 patients with *BRCA*wt 9 *BRCA1*m 2 *BRCA2*m	Pembrolizumab	Niraparib	**ORR**: 18% (11–29) 3 CR, 8 PR **DCR**: 65% (54–75) 28 SD	**PFS**: 3.4 mos (2.1–5.1) OS: Not reported
MEDIOLA NCT02734004 Single arm Phase II Drew *et al*. 2019 ESMO Congress [15]	32 evaluable patients Recurrent setting Platinum-sensitive 22 *BRCA1*m 10 *BRCA2*m	Durvalumab	Olaparib	**ORR**: 71.9% (53.25–86.25) 7 CR, 16 PR **DCR** at **28 weeks**: 65.6% (49.6–79.4)	**PFS**: 11.1 mos (8.2–15.9) **OS**: Not reported
KEYNOTE-100 NCT02674061 Single arm Phase II Matulonis *et al*. 2020 ASCO Meeting [8]	376 patients Recurrent setting Cohort A (*n* = 285): 0–2 prior lines of treatment Cohort B (*n* = 91): 3–5 prior lines of treatment *BRCA*m status: Not reported	Pembrolizumab	N/A	ORR combined **Total**: 8.5% (5.9–11.8) **CPS ≥ 10**: 13.8% (6.5–24.7) CPS ≥ 1: 8.0% (4.2–13.6) **CPS < 1**: 7.3% (3.4–13.3) ORR cohort A **Total**: 8.1% (5.2–11.9) **CPS ≥ 10**: 11.6 (3.9–25.1) **CPS ≥ 1**: 6.9 (2.8–13.8) ORR cohort B **Total**: 9.9% (4.6–17.9) **CPS ≥ 10**: 18.2 (5.2–40.3) **CPS ≥ 1**: 10.2 (3.2–22.2) DCR combined **Total**: 22.1% (18.0–26.6) **CPS ≥ 10**: 27.7% (17.3–40.2) **CPS ≥ 1**: 24.0% (17.4–31.6) DCR cohort A **Total**: 22.1% (17.4–27.4) **CPS ≥ 10**: 25.6% (13.5–41.2) **CPS ≥ 1**: 24.8% (16.7–34.3) DCR cohort B **Total**: 22.0% (14.0–31.9) **CPS ≥ 10**: 31.8% (13.9–54.9) **CPS ≥ 1**: 22.4% (11.8–36.6) None of the above subgroup analyses reached statistical significance (*p* < 0.05)	PFS cohort A **Total**: 2.1 mos (2.1–2.2) **CPS ≥ 10**: 2.1 mos (2.1–4.2) **CPS ≥ 1**: 2.1 mos (2.1–2.8) PFS cohort B **Total**: 2.1 mos (2.1–2.6) **CPS ≥ 10**: 2.1 mos (2.0–8.3) **CPS ≥ 1**: 2.1 mos (2.1–3.3) OS cohort A **Total**: 18.7 mos (17.0–22.5) **CPS ≥ 10**: 21.9 mos (12.9–26.8) **CPS ≥ 1**: 20.6 mos (15.2–23.2) OS cohort B **Total**: 17.6 mos (13.3–24.4) **CPS ≥ 10**: 24.0 mos (14–NR) **CPS ≥ 1**: 20.7 mos (13.6–27.4) None of the above subgroup analyses reached statistical significance (*p* < 0.05)
JAVELIN-200 NCT02580058 Randomized Phase III Pujade-Lauraine *et al*. 2019 SGO Meeting [6]	566 evaluable patients Recurrent setting 424 platinum-resistant 142 platinum-refractory Avelumab + PLD (*n* = 188) Avelumab alone (*n* = 188) PLD alone (*n* = 190) *BRCA*m status: Not reported	Avelumab	PLD	**ORR combined**: 13.3% (8.8–19.0) **ORR avelumab alone**: 3.7% (1.5–7.5) **ORR PLD alone**: 4.2% (1.8–8.1)	PFS combined **Total**: 3.7 mos (3.3–5.1) **PD-L1+**: 3.7 mos (2.2–5.6) **PD-L1–**: 3.9 mos (1.9–5.5) PFS avelumab alone **Total**: 1.9 mos (1.8–1.9) **PD-L1+**: 1.9 mos (1.8–2.3) **PD-L1–**: 1.8 mos (1.8–1.9) PFS PLD alone **Total**: 3.5 mos (2.1–4.0) **PD-L1+**: 1.9 mos (1.9–3.6) **PD-L1–**: 3.7 mos (3.2–5.5) OS combined: **Total**: 15.7 mos (12.7–18.7) **PD-L1+**: 18.4 mos (13.7–22.0) **PD-L1-**: 12.7 mos (7.8–18.7) OS avelumab alone: **Total**: 11.8 mos (8.9–14.1) **PD-L1+**: 13.7 mos (9.6–NE) **PD-L1–**: 10.5 mos (6.8–15.3) OS PLD alone: **Total**: 13.1 mos (11.8–15.5) **PD-L1+**: 13.8 mos (10.5–17.7) **PD-L1–**: 13.1 mos (10.9–15.7) None of the above subgroup analyses reached statistical significance (*p* < 0.05)
IMagyn050 NCT03038100 Randomized Placebo-controlled Phase III Moore *et al*. 2020 ESMO Congress [16]	1301 patients Upfront setting *BRCA*m status: Not reported	Atezolizumab	Chemotherapy + Bevacizumab	Not reported	**PFS (ITT)**: 19.5 vs. 18.4 mos (HR 0.92; 95% CI 0.79–1.07) **PFS (PD-L1+)**: 20.8 vs. 18.5 mos (HR 0.80; 95% CI 0.65–0.99) None of the above subgroup analyses reached statistical significance (*p* < 0.05)

Abbreviations: *BRCA*wt, BRCA wild type; *BRCA1*m, *BRCA1* mutation; *BRCA2*m, *BRCA2* mutation; CI, confidence interval; CPS, combined positive score; CR, complete response; DCR, disease control rate; HR, hazard ratio; ITT, intent-to-treat; mos, months; N/A, not applicable; NE, not estimable; NR, not reached; ORR, objective response rate; OS, overall survival; PFS, progression-free survival; PLD, pegylated liposomal doxorubicin; PR, partial response; SD, stable disease.

Lastly, ascertaining the optimal time for introduction of ICB in the life cycle of ovarian cancer treatment is a matter of intense scientific scrutiny, and attention must be paid to predictive biomarkers and clinical benefit previously discussed. Clinical trials in the upfront setting often operate under the notion that there are more immunoreactive cells earlier in the course of disease, rendering ICB more effective. However, whether this assumption is correct remains to be seen. Preliminary results from IMagyn050 presented at the 2020 ESMO meeting showed no improvement in PFS with the anti–PD-L1 antibody atezolizumab in the intent-to-treat or PD-L1–positive population in the upfront setting, calling into question both the first-line setting as well as PD-L1 as a predictive biomarker [16]. Another key consideration is primary platinum resistant/refractory disease present in about 20% of newly diagnosed patients, in which patients progress within 6 months after completing first-line platinum-based therapy or fail to respond to first-line treatment [17]. It is likely that at least some degree of the lack of response to ICB in the upfront setting is attributable to this intrinsic resistance to chemotherapy, which has unique molecular and clinical characteristics. Furthermore, ovarian carcinoma with a “cold” immunosuppressive TME may preclude any response to immunotherapy at all [18]. Therefore, the conventional approach of incorporating ICB based on the presumed favorable immunostimulatory TME may not be applicable to a sizable proportion of patients in the upfront setting. Separate multi-cohort and multi-arm treatment trial approaches for primary platinum resistant/refractory disease, as well as a closer investigation into biomarkers and the TME of this unique subgroup, are warranted.

Immune checkpoint inhibitors, though promising in several cancers, have shown relatively limited activity thus far in ovarian cancer. The full significance of the TME in determining response to immunotherapy combination approaches has yet to be elucidated, and incorporating fresh paired tumor biopsies into clinical trials is an important step in that direction. Future studies must prioritize identification of biomarkers of response and resistance in order to better guide where in the treatment life cycle ICB therapies will yield greatest effect, as well as which population will most benefit. Such correlative studies are crucial in bridging the gap between preclinical promise and clinical success, ultimately moving the needle forward in ovarian cancer treatment.
